# Increased serum levels of MIC1/GDF15 correlated with bone erosion in spondyloarthritis: A pilot study

**DOI:** 10.1097/MD.0000000000013733

**Published:** 2018-12-21

**Authors:** Yingyu Song, Yang Cui, Xiao Zhang, Haobo Lin, Guangfeng Zhang, Hui Zeng, Yonghan Zeng

**Affiliations:** a Department of Rheumatology, Guangdong General Hospital, Guangdong Academy of Medical Sciences; b Southern Medical University; c Medical Imaging Centre, Guangdong General Hospital, Guangdong Academy of Medical Sciences, Guangzhou, Guangdong, China.

**Keywords:** bone, erosion, macrophage inhibitory cytokine 1/Growth differentiation factor-15 (MIC1/GDF15), magnetic resonance imaging, Spondyloarthritis (SpA)

## Abstract

**Introduction:**

To assess the association between growth differentiation factor-15 (GDF15) and radiographic features including bone marrow edema and bone erosion in Spondyloarthritis (SpA).

**Methods:**

Patients with SpA (n = 120) receiving treatment in the Guangdong General Hospital, China, between August 2012 and December 2016 were retrospectively included. Serum of patients and healthy controls (n = 30) were collected and GDF15 levels were measured using ELISA. Inflammation was assessed by C-reactive protein (CRP), and magnetic resonance imaging (MRI) of the sacroiliac joint using Spondyloarthritis Research Consortium of Canada score and a method of dichotomy to assess fat metaplasia, bone erosion, and ankylosis. Radiographs of the pelvis were scored using the modified New York (mNY) score.

**Results:**

Serum GDF15 levels were higher in SpA patients compared to controls (503.52 ± 222.92 vs. 190.86 ± 104.18 pg/mL, *P* < .0001). Patients who suffered from bone erosion on MRI had higher levels of GDF15 (525.72 [186.33, 801.62]vs. 428.06 [255.15, 670.98] pg/mL, *P* = .0375). There was a positive correlation between serum GDF15 and CRP (r = 0.5442, *P* < .0001). Moreover, GDF15 levels were related to CRP levels (r = 0.5658, *P* < .0001) in those X-ray scores were III, according to 1984mNY criteria. Receiver operating characteristic (ROC) analysis showed that GDF15 levels above 501.98pg/mL could predict presence of bone erosion on MRI.

**Conclusion:**

The present study suggested that serum GDF15 levels are higher in SpA patients than in healthy controls. The GDF15 level was correlated with CRP and may be a surrogate biomarker in bone erosion.

## 1 Introduction

Spondyloarthritis (SpA) is an inflammatory disorder that typically affects spine and sacroiliac joint. With the development of magnetic resonance imaging (MRI) technique, the diagnosis and assessment of SpA have been improved greatly. However, MRI examination is costly and interpretation of MRI images requires specialized training. There is an urgent need to discover useful serum biomarkers which can add values to radiographic diagnostic techniques.

Several molecules are involved in new bone formation resulting in structural damage in patients with SpA. Sclerostin (SOST) is a glycoprotein secreted by osteocytes in bone, and it inhibits Wnt signaling pathway, leads to decreased bone formation.^[1]^ A study revealed that the serum levels of SOST were found to be elevated in patients with SpA with high disease activity.^[2]^ However, SOST neither elevated in SpA with low disease activity nor it linked to greater structural damage.^[3]^

Growth differentiation factor (GDF)-15, also called as macrophage inhibitory cytokine 1 (MIC1), a member of the transforming growth factor-beta superfamily of cytokines, plays an important role in cell growth and differentiation.^[4]^ The GDF15 regulates inflammatory pathway and promotes inflammation.^[5]^ Lambrecht S et al ^[6]^ found that the patients with SpA had a significantly higher concentration of GDF15 in the synovial fluid. Thus, a local production of GDF15 in the synovial joint may result in elevated levels of GDF15 in serum. In contrast, in another study, it was found that GDF15 was dramatically secreted after activation of macrophage cells by tumor necrosis factor-alpha (TNF-α) and transforming growth factor-beta (TGF-β), wherein GDF15 played an anti-inflammatory role in inflammation process ^[7]^. In our previous study, an increased expression of TNF-α and TGF-β mRNAs was noted along with significant infiltration of inflammatory cells in the sacroiliac joint.^[8]^ Another study revealed that in bone, GDF15 was secreted from osteocytes under hypoxic condition which promoted osteoclastic differentiation and reduced bone volume.^[9]^ Therefore, we hypothesized that GDF15 could be involved in inflammation and bone erosion in SpA.

The present study aimed to compare the GDF15 serum levels between patients with SpA and healthy controls and explain the importance of GDF15 as a serum biomarker for SpA along with radiographic and MRI parameters.

## 2 Patients and methods

### 2.1 Patients

In this retrospective observational study, patients with SpA (n = 120) receiving treatment in the Guangdong General Hospital, Guangzhou, Guangdong, China, between August 2012 and December 2016 were included. The study eligible criteria were as follows: met the Assessment of SpondyloArthritis international Society (ASAS) classification criteria 2009; had MRI data of the sacroiliac joint; serum samples collected and preserved at -80°C; and without any other infectious disease or organ function failure. In the control group (n = 30), healthy volunteers (who were working as paramedics at the rheumatology department) of ages between 20 and 38 years old were enrolled; all of them did not have any rheumatic diseases or inflammatory back pain.

The study was approved by the Ethics Committee of Guangdong General Hospital, China. Informed written consent from patients was waived as it was a retrospective study. (Ethical approval number: 2015327H)

### 2.2 Assessment of inflammatory and serum GDF15

Before MRI examination, at fasting state for more than 6 hours, patient's blood samples were obtained and centrifuged immediately. Serum was separated and stored at -80°C. The serum concentration of GDF15 and SOST were determined using an enzyme-linked immunosorbent assay kit (R&D systems, Minneapolis), according to the recommendations of the manufacturers. Other parameters including erythrocyte sedimentation rate (ESR) (mm/h) and C-reactive protein (CRP) (mg/L) were obtained from patient's medical records retained by Hospital Information Management System. The levels of ESR above 15 mm/h and CRP above 8 mg/L were considered abnormal. We used Bath Ankylosing Spondylitis Disease Activity (BASDAI), Bath Ankylosing Spondylitis Functional Index (BASFI), and Ankylosing Spondylitis Disease Activity Score (ASDAS) to assess severity and function of patients.

### 2.3 Radiographic and MRI parameters

The bone marrow edema was scored based on Spondyloarthritis Research Consortium of Canada (SPARCC) scoring system.^[10]^ In brief, this scoring method is based on the assessment of increased signal on T2 with fat suppression or short tau inversion recovery (STIR) sequences denoting bone marrow edema on oblique coronal slices of the sacroiliac joint. All such signal changes within the iliac bone and sacrum up to the sacral foramina are scored on 6 consecutive slices through the sacroiliac joint. These slices are selected based on the SPARCC protocol. Sacral interforaminal bone marrow STIR signal forms the reference for determination of increased signal in the sacroiliac joint. Each sacroiliac joint is divided into 4 quadrants, and the presence of increased STIR signal in each of these 4 quadrants is recorded in each of the 6 slices, giving a maximum score of 48. The presence of a lesion exhibiting either intense or depth signal anywhere within each sacroiliac joint of the 6 slices is given an additional score, bringing the total score to 72. The method was same with a previous report.^[11]^

Erosion was defined as loss of marrow signal on T1 sequence (T1-SE) together with a defect in the overlaying cortical bone.^[12]^ The severity of radiographic damage was assessed by 1984 Modified New York (mNY) criteria for ankylosing spondylitis on X-ray images. Radiographs of the pelvis were performed and scored by 2 rheumatologists (with 4 and 14 years’ work experience respectively) with extensive experience in musculoskeletal X-ray assessment. Assessments of MRI were also scored by 2 radiologists (with 12 and 30 years’ work experience). If there was disagreement between the assessors, the case was referred to 3rd party (with 24 years working experience) and reached a consensus.

### 2.4 Statistical analysis

Data for continuous variables fulfilled normal distribution were presented as mean ± standard deviation and abnormal distribution variables were presented a median (10 and 90% quartiles). The Kolmogorov–Smirnov test was used to assess the normality of distribution of continuous variables. Independent *t* test or Mann–Whitney *U* test was used to examine the differences in continuous variables between 2 groups. Data for categorical variables were presented as number (percentage). Spearman's coefficient of correlation was used for bivariate correlations. Cut-off value for GDF15 was selected using receiver operating characteristic (ROC) analysis. Statistical analysis was performed using SPSS for Windows version 20.0 (IBM, New York).

## 3 Results

### 3.1 Characteristics of the patients with SpA

The patients with SpA included 104 men and 16 women. Among these patients, 113 (94.20%) were HLA-B27 positive. According to clinical manifestations, X-ray results, and MRI features, 92 patients fulfilled 1987 mNY criteria. Twenty eight patients did not fulfill the criteria and were classified as non-radiographic SpA (nr-SpA) according to 2009 ASAS criteria. The median duration of symptoms was 2.00 years. The disease duration was not statistically significant between nr-SpA and ankylosing spondylitis (AS) patients (2.00 [0.50, 10.00] vs. 2.00 [0.50, 6.10] years, *P* = .4008). The demographic characteristics of patients were summarized in Table 1.

**Table 1:**
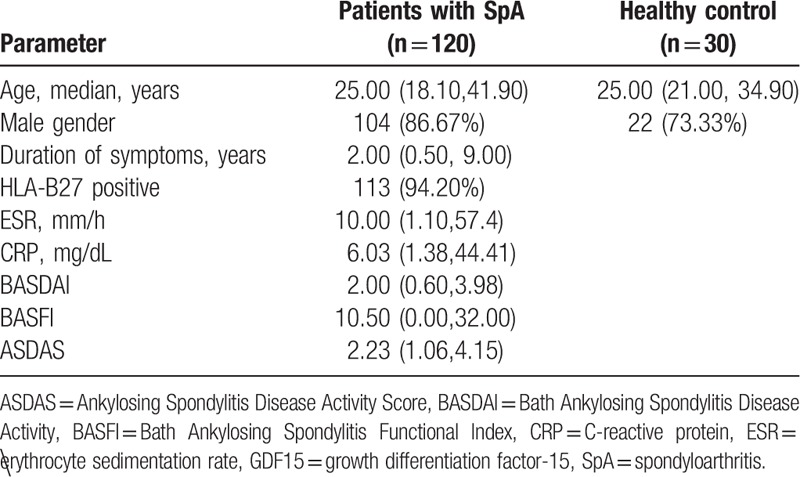
Demographic, clinical, laboratory and radiographic data of patients and healthy controls.

### 3.2 GDF15 and SOST serum levels of patients with SpA and controls

**Figure 1:**
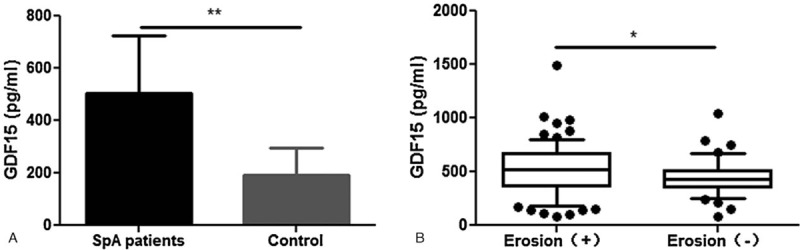
Growth differentiation factor-15 (GDF15) levels rise in Spondyloarthritis (SpA) patients than normal control (**A**), but mainly in those with erosion (**B**).^∗^*P* < .05 ^∗∗^*P* < .01. GDF15 = growth differentiation factor-15, SpA = Spondyloarthritis.

**Figure 2:**
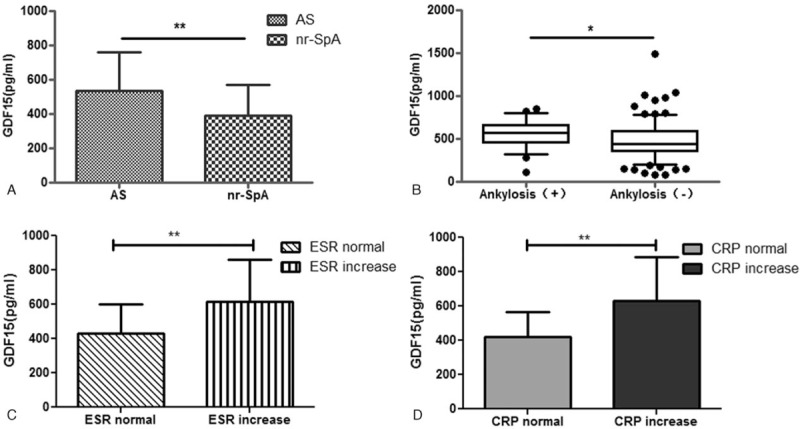
Growth differentiation factor-15 (GDF15) levels showed differences by subgroups of AS and nr-SpA (**A**) or ankylosis (**B**), or ESR (**C**), CRP (**D**). ^∗^*P* < .05 ^∗∗^*P* < .01. CRP = C-reactive protein, ESR = erythrocyte sedimentation rate, GDF15 = growth differentiation factor-15.

The GDF15 levels were significantly higher in patients with SpA compared to control group (503.52 ± 222.92 vs. 190.86 ± 104.18 pg/mL, *P* < .0001, Fig. 1**A**). Patients who suffered from bone erosions on MRI had much higher levels of GDF15 compared to those without erosions (525.72 [186.33, 801.62] vs. 428.06 [255.15, 670.98] pg/mL, *P* = .0375, Fig. 1**B**). Meanwhile, the levels of GDF15 were different between subgroups of SpA according to other categorical variables (CRP, ESR, and ankylosis) presented in Fig. 2. No significant difference of GDF15 was found between patients with or without fat metaplasia. We also found that levels of GDF15 were significantly higher in AS patients when compared to nr-SpA patients (537.38 ± 225.14 vs. 392.27 ± 177.35 pg/mL, *P* = .0022, Fig. 2**A**). There were no significantly differences of SOST levels between AS and nr-SpA patients, but the former had lower SOST levels (328.41 ± 138.12 vs. 387.07 ± 162.76 pg/mL, *P* = .0618).

### 3.3 Association of serum GDF15 levels with clinical features, inflammatory, and radiographic variables in SpA patients

The correlations between serum GDF15 levels and serum levels of other factors involved in inflammation and bone formation are shown in Table 2. The correlation analysis showed that there was a positive correlation between serum GDF15 and CRP (r = 0.5442, *P* < .0001, Table 2). According to the severity of X-ray, GDF15 levels were positively and significantly correlated with CRP levels (r = 0.5658, *P* < .0001) whose X-ray scores were III degree. Levels of ESR and CRP were positively correlated with GDF15 (r > 0.3, *P* < .0001, Table 2). In addition, GDF15 had an association with levels of BASDAI, BASFI, and ASDAS scores (Table 2). No significant correlations were found between GDF15 levels and SOST, SPARCC score.

**Table 2:**
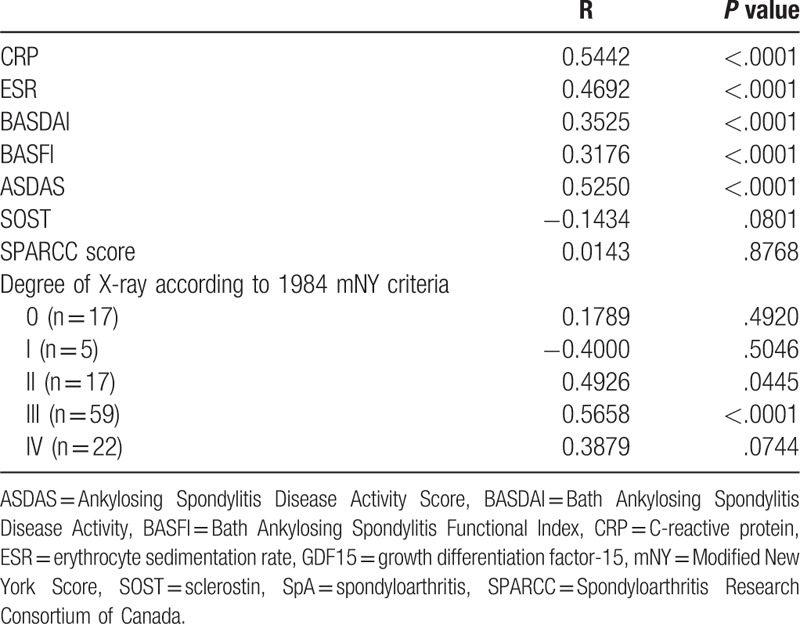
Correlation coefficients (r) of serum GDF15 levels with ESR, CRP, BASDAI, BASFI, ASDAS, SOST, and SPARCC scores.

### 3.4 Prediction of no bone erosion using serum GDF15 levels

**Figure 3:**
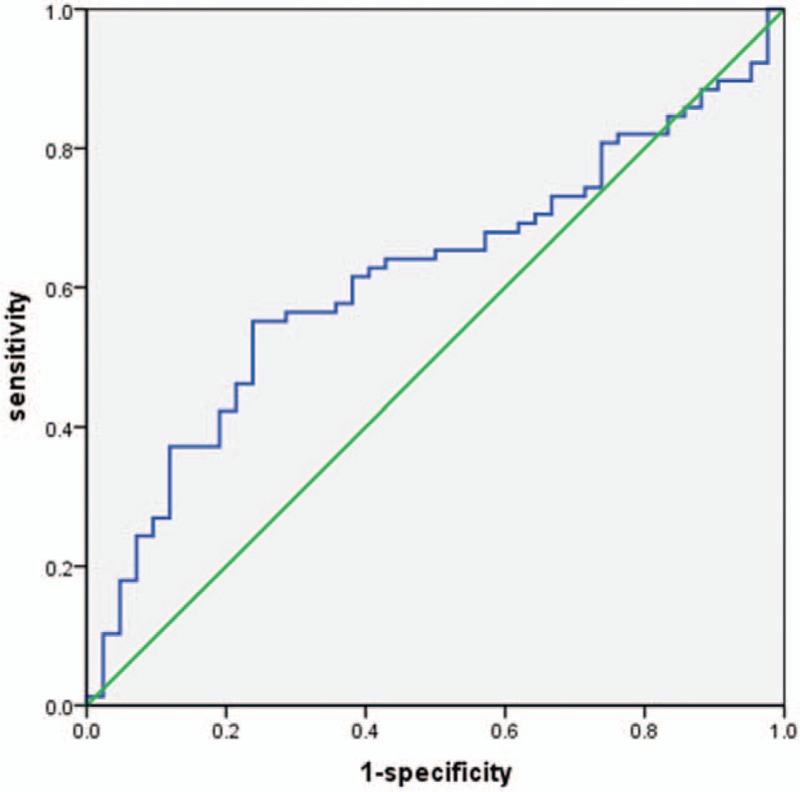
Receiver operating characteristic (ROC) of growth differentiation factor-15 (GDF15) level for predicting bone erosion. GDF15 = growth differentiation factor-15, ROC = receiver operating characteristic.

The ROC analysis was adopted to predict patients who suffered from bone erosion (Fig. 3). Cut-off value for GDF15 was 501.98pg/mL. An area under the ROC curve was 0.62 and the sensitivity and specificity were 55.13% and 76.19%, respectively.

## 4 Discussion

The SpA is an inflammatory arthritis disease and its pathologic changes include an initial inflammation to bone destruction, followed by fat metaplasia and new bone formation. Various inflammatory cytokines such as TNF-α, interleukin (IL)-1, IL-6, and IL-23 participate in the inflammatory bone destruction. In the present study, GDF15 levels in patients with SpA were compared and found that GDF15 level was higher in patients who have bone erosion. As far as we know, this is the 1st study focusing on relationship between serum GDF15 levels and MRI changes in SpA.

It is well known that bone erosion is a predominant pathologic feature in rheumatoid arthritis (RA) and GDF15 can predict the presence of severe disease and bone erosion in RA.^[13]^ Moreover, under hypoxia, osteocytes could secret GDF15 to promote osteoclastogenesis.^[9]^ Compared to patients with RA, it was found that GDF15 levels were lower in SpA.^[5,13]^ In the present study, it was found that GDF15 levels were higher in patients with bone erosion compared to those without bone erosion. It was speculated that this may due to 2 reasons as follows. First, GDF15 could function as an anti-inflammatory factor induced by inflammation ^[14–16]^ leading to bone destruction. Second, inflammatory cytokine TNF-α can promote GDF15 expression and secretion.^[17]^ Hence, it was assumed that the patients with higher GDF15 level might have higher TNF-α level which was more likely to suffer from bone erosion. We speculated that GDF15 was a surrogate biomarker in bone metabolism. There is an apparent need for further study to explore the role of GDF15 in SpA pathologic process.

According to radiographic features, axial SpA is commonly divided into nr-axSpA and AS. In the present study, serum GDF15 levels of nr-SpA patients were much lower than AS patients. When taking severity of X-ray into consideration, it was found that GDF15 was positively correlated with CRP level, especially in III degree radiographic destruction. Inflammatory factors could stimulate bone formation pathways in the pathology process of SpA. Activation of these pathways lead to pathological ossification ,^[18]^ and thus it may explain that the levels of inflammatory factors are all elevated in AS patients.

Radiographic as well as laboratory results are both critical for disease diagnosis and monitoring the progress of the disease. The MRI scans of sacroiliac joint shows bone edema reflecting inflammatory pathological changes and SPARCC score system has been widely accepted as a useful method to assess degree of bone edema. In the present study, SPARCC scores of patients were high. However, no evidence for relationship between high SPARCC scores and GDF15 levels was found. Hence, GDF15 could be a new meaningful biomarker for inflammation along with CRP. It was noted that bone marrow edema could be evident in patients with nonspecific back pain compared to healthy controls.^[19]^ Structural lesions are considered critical in the diagnosis of SpA recently. Weber et al ^[19]^ found that bone marrow edema as well as bone erosion together accounts for the structural changes identified in the MRI of nr-SpA patients. These findings remind us to focus on bone marrow edema and bone erosions at the same time. However, it was not confirmed in the present study that GDF15 in combination with bone marrow edema could promote positive sacroiliac joint criteria which require further extensive investigation.

The SOST is a molecule which inhibits the Wnt signaling pathway, leading to decreased bone formation. It was reported that low level of SOST was related to new syndesmophyte formation.^[20]^The SOST could be responsible for the suppression of Wingless protein-3a and dikkopf-1 protein levels in SpA with high disease activity.^[3]^ However, there are contradictory results regarding the association of SOST in SpA patients.^[21–23]^ In the present study, no difference between the 2 groups was noted. This may attribute to small sample size of patients and short-term duration of study.

The present study has several limitations. First of all, this is a single-center, retrospective, observational study with limited patient data. Hence, the small sample size can have profound effects on the study outcome and may limit the statistical power of the results. Secondly, we just focused on the relationship between GDF15 and clinical features of SpA; however, this was not a cause and effect outcome. The exact role of GDF15 in SpA needs to be verified in further large-scale experimental and clinical trials.

In conclusion, higher GDF15 levels in patients with SpA are positively correlated to CRP, and it may play a role in bone erosion. The present study findings need to be verified further with larger clinical trials with long-term follow-up.

## Author contributions

**Conceptualization:** Yingyu Song, Hui Zeng.

**Data curation:** Yingyu Song, Xiao Zhang, Haobo Lin, Guangfeng Zhang, Hui Zeng, Yonghan Zeng.

**Formal analysis:** Yang cui, Yingyu Song, Guangfeng Zhang, Hui Zeng, Yonghan Zeng.

**Funding acquisition:** Yang cui.

**Investigation:** Yingyu Song, Haobo Lin, Guangfeng Zhang, Hui Zeng, Yonghan Zeng.

**Methodology:** Haobo Lin.

**Project administration:** Yang cui.

**Software:** Xiao Zhang.

**Writing – original draft:** Yingyu Song.

**Writing – review & editing:** Yang cui, Yingyu Song, Haobo Lin.
